# Use of CompEx in eosinophilic patients with severe, uncontrolled asthma on benralizumab

**DOI:** 10.1183/23120541.01025-2023

**Published:** 2024-03-18

**Authors:** Clare Bolton, Tim Harrison, Njira Lugogo, Anne Fuhlbrigge, Ian Hirsch, Thomas Bengtsson, Stefan Peterson, Martin Sidaway, Esther Garcia Gil, Malin Fagerås, Carla A. Da Silva

**Affiliations:** 1 BioPharmaceuticals Medical, AstraZeneca, Cambridge, UK; 2 Nottingham Respiratory NIHR BRC, University of Nottingham, Nottingham, UK; 3 Division of Pulmonary and Critical Care, Department of Medicine, University of Michigan, Ann Arbor, MI, USA; 4 Pulmonary Sciences and Critical Care, Department of Medicine, University of Colorado School of Medicine, Aurora, CO, USA; 5 Late-stage Vaccine and Immune Therapies, BioPharmaceuticals R&D, AstraZeneca, Cambridge, UK; 6 StatMind Statistical and Mathematical Modelling, Innovation and Design AB, Lund, Sweden; 7 Early Respiratory and Immunology Clinical Development, BioPharmaceuticals R&D, AstraZeneca, Gothenburg, Sweden; 8 GMA Respiratory & Immunology, AstraZeneca, Barcelona, Spain; 9 BioPharmaceuticals Medical, AstraZeneca, Gothenburg, Sweden

## Abstract

**Background:**

CompEx Asthma, a composite end-point for asthma exacerbations, captures clinically relevant, diary-based acute worsening events (AWEs) (defined as deterioration in daily peak expiratory flow concurrent with deterioration in asthma symptoms and/or rescue therapy use) and severe exacerbations (SevEx) (defined by American Thoracic Society/European Respiratory Society guidelines). We hypothesised that CompEx and SevEx would show similar benralizumab treatment effects and correlations to blood eosinophil counts in patients with severe asthma.

**Methods:**

This *post hoc* analysis of pooled 12-month data from two phase 3 studies included patients aged ≥16 years with severe, uncontrolled asthma who were randomised to benralizumab 30 mg or placebo. Annualised event rates were analysed using a negative binomial model. The impact of blood eosinophil count on treatment effect was assessed.

**Results:**

Among patients with a blood eosinophil count ≥300 cells·µL^−1^ (n=913), benralizumab reduced the annualised event rate *versus* placebo for CompEx (1.57 *versus* 2.57; risk ratio 0.61, 95% CI 0.53–0.70, p<0.001), SevEx (0.94 *versus* 1.55; risk ratio 0.60, 95% CI 0.52–0.70, p<0.001) and AWE (0.92 *versus* 1.57; risk ratio 0.59, 95% CI 0.48–0.72, p<0.001), with greater treatment effects observed for higher blood eosinophil counts. In patients with blood eosinophil count ≥300 cells·µL^−1^, benralizumab was associated with shorter median event duration (CompEx: 10.5 days *versus* 17.0 days; SevEx: 10.0 days *versus* 15.0 days; AWE: 5.0 days *versus* 6.0 days).

**Conclusions:**

Benralizumab reduced the risk of CompEx events with treatment effects similar to those for SevEx and AWEs across a range of blood eosinophil counts. Use of CompEx supports the evaluation of benralizumab and other novel drugs in clinical studies.

## Shareable abstract

**In a *post hoc* analysis of data from two phase 3 benralizumab trials, the treatment effect on the composite end-point CompEx Asthma, or its components, mimicked that observed for severe asthma exacerbations, across a range of blood eosinophil counts** https://bit.ly/3O1Mc6S

## Introduction

Asthma is a chronic inflammatory disease of the lungs characterised by recurring respiratory symptoms and fluctuating airflow obstruction [1–3]. It is thought to affect an estimated 339 million people worldwide [4], of whom up to 10% have severe disease [5]. Prevention of severe exacerbations (SevEx) is a major goal of asthma treatment [1].

The American Thoracic Society (ATS)/European Respiratory Society (ERS) guidelines define SevEx as the use of oral systemic corticosteroids (OCS) or an increase from a stable maintenance dose for at least 3 days, or a hospitalisation or an emergency department visit due to asthma that required OCS treatment [6]. However, this definition relies on patients’ subjective perceptions of their own airway symptoms, and patient and physician perceptions of the need for OCS. This can result in OCS being overprescribed in some cases and not being prescribed when needed in other cases.

An extension of the definition of SevEx events in clinical trials has been described recently. In a *post hoc* analysis published in 2017, Fuhlbrigge *et al*. [7] developed a novel, composite end-point for asthma exacerbations (CompEx Asthma, hereafter referred to as CompEx) combining SevEx and acute worsening events (AWEs). AWEs are diary card events defined by deteriorations in peak expiratory flow (PEF) concurrent with increases in rescue therapy use and/or deteriorations of daily asthma symptoms. This end-point therefore includes additional events that are objectively defined, yet are not necessarily treated with OCS, and captures both clinical and physiological patient-reported outcomes. While rates of SevEx can provide insights into treatment efficacy in patients with severe eosinophilic asthma, they are seldom used as end-points in early clinical development trials due to their infrequent nature and the need for large and lengthy trials to provide sufficient statistical power. The development of CompEx allows the design of shorter and smaller clinical trials that are able to reproduce treatment effects observed for SevEx [7]. Furthermore, seasonal variations in asthma exacerbation rates observed for SevEx are also observed for CompEx, while month-by-month variation and variation across different geographical regions are reduced when using CompEx as an end-point [8]. Thus, the inclusion of AWEs in CompEx may help to reduce overall variability in event rates, with increased sensitivity when SevEx rates are low [8].

SevEx events, while infrequent overall, are most common in patients with severe, uncontrolled asthma and eosinophilia [6, 9]. Increased numbers of eosinophils in the airways are also associated with greater symptom burden and decreased lung function [10, 11]. In this population (Global Initiative for Asthma (GINA) Steps 4–5), GINA currently recommends add-on biological therapies, including those against interleukin-5, a cytokine involved in eosinophil development, activation, proliferation and survival [1].

The efficacy and safety of benralizumab, an anti-interleukin-5 receptor-α monoclonal antibody that rapidly depletes eosinophils [12], was demonstrated in the SIROCCO (ClinicalTrials.gov: NCT01928771) and CALIMA (ClinicalTrials.gov: NCT01914757) phase 3 trials in patients with severe, uncontrolled asthma who had experienced at least two asthma exacerbations in the previous year; a greater treatment effect was observed in the subgroup of patients with blood eosinophil counts ≥300 cells·µL^−1^ [13, 14]. Both studies reported significant improvements in annual SevEx rates, lung function and symptom control with benralizumab *versus* placebo [14].

In this *post hoc* analysis of data from the SIROCCO and CALIMA trials, we aimed to determine the effect of benralizumab on the risk of CompEx events and its individual components (SevEx and AWEs), and its relationship to patients’ baseline blood eosinophil counts.

## Methods

### Study design and data collection

The study design and inclusion of participants for the SIROCCO and CALIMA phase 3 clinical trials have been previously described in detail [13, 14]. Both studies were performed in accordance with the Declaration of Helsinki, International Conference on Harmonisation of Technical Requirements for Registration of Pharmaceuticals for Human Use and Good Clinical Practice guidelines, and the ethics committee at each participating site.

In this *post hoc* analysis, we included patients from the benralizumab 30 mg 8-weekly and placebo arms of the SIROCCO and CALIMA phase 3 clinical trials, because this is the approved dosing schedule. For consistency with the patient populations assessed in previous CompEx publications [7], only patients aged ≥16 years were included in our analysis. Data from the benralizumab 4-weekly arms were not included, because every 8 weeks is the approved dose of benralizumab in severe eosinophilic asthma [15, 16]. Data from the medium- and high-dose inhaled corticosteroid (ICS)/long-acting β_2_-agonist (LABA) groups were pooled for all analyses. Additionally, we focused our analyses on the subgroup of patients with baseline blood eosinophil counts ≥300 cells·µL^−1^; this was consistent with patients included in the primary analyses of SIROCCO and CALIMA [13, 14] and with current labelling [17]. However, we also evaluated the impact of baseline blood eosinophil count on the benralizumab treatment effect over the entire spectrum of blood eosinophil counts.

### Definitions of CompEx, SevEx and AWE

CompEx is a composite measure of exacerbations that captures both clinically relevant, diary-based AWEs and SevEx events [7]. In line with SIROCCO and CALIMA, SevEx events were defined as requiring ≥3 days of OCS treatment and/or hospitalisation or an emergency department visit due to asthma that required OCS. An AWE has been previously defined in detail [7, 8]. Briefly, AWEs comprise deterioration in PEF concurrent with increased rescue therapy use and/or deterioration of asthma symptoms in the morning and evening. AWEs were captured when either 1) at least two diary variables (one of which must be PEF) reached a prespecified threshold change from baseline over at least two consecutive days, or 2) when worsening greater than a certain magnitude (slope; daily rate of change) in all diary card variables occurred over ≥5 days, combined with a threshold change of at least one variable.

Each CompEx event was defined as the incidence of either an AWE or SevEx event, whichever occurred first, with the start/end of an event marked as the first/last day that the criteria for either a diary event or SevEx were fulfilled. Events starting or ending within 7 days of each other were considered a single event.

### Statistical analysis

The number of each event type was summarised for each study and treatment arm. Analyses of the annualised event rate (AER) and the time to first event are presented for the pooled population and for the SIROCCO and CALIMA studies separately.

A negative binomial model was used to analyse the number of events (CompEx, AWEs or SevEx), adjusted for treatment, region, number of previous exacerbations (≤2, ≥3) and use of maintenance OCS (yes/no) as fixed factors, and the natural logarithm of time in the study as an offset. For the pooled analyses, “study” was included as an additional fixed factor. Estimated AER and treatment effect are expressed as a risk ratio for the comparison between benralizumab and placebo, together with 95% confidence intervals (CIs) and the associated two-sided p-value. Cox proportional hazards models, adjusted for the same factors as the negative binomial model, were used for time-to-first-event analyses, which are presented as Kaplan–Meier plots, and from which median event durations were determined. Patients with no events were censored at the last day in the treatment period. The treatment effect is expressed as a hazard ratio (HR) between benralizumab and placebo, along with 95% CIs and two-sided p-values.

A cumulative approach was used to investigate the predictive and prognostic properties of baseline blood eosinophil counts, using cut-off levels that cover the spectrum of eosinophil counts, from a minimum cut-off of 70 cells·µL^−1^ to a maximum cut-off of 800 cells·µL^−1^. Patients were divided into a lower stratum (≤cut-off) and a higher stratum (>cut-off) at any given cut-off point, and analyses from different cut-offs were combined to determine the relationship between baseline eosinophil count cut-off and size of effect for CompEx events (and for its individual components). For this analysis, data from SIROCCO and CALIMA were pooled. Risk ratios from each analysis were plotted *versus* the cut-off level, showing the change in effect when extending the eosinophil range upwards (lower stratum, left to right) or downwards (higher stratum, right to left). The risk ratio from the analysis of the full population was included as a reference to show the convergence point of the curves.

## Results

### Patients

A total of 1612 patients from SIROCCO (n=768) and CALIMA (n=844) aged ≥16 years were included. Overall, 64% of patients were female, the mean age was approximately 50 years and the first asthma diagnosis was at approximately 31 years. 63% of patients had two exacerbations, 20% had three, 7% had four and 9% had five or more exacerbations in the previous year; 14% received maintenance OCS therapy. The median baseline blood eosinophil count was 330 cells·µL^−1^ for those receiving benralizumab and 360 cells·µL^−1^ for those receiving placebo, and 913 patients (SIROCCO n=418; CALIMA n=495) had baseline blood eosinophil counts ≥300 cells·µL^−1^. Around 92% of all patients were receiving high-dose ICS-containing ICS/LABA therapies (table 1).

**TABLE 1:** Baseline demographics in patients aged ≥16 years from the SIROCCO and CALIMA studies (pooled data), for patients with a baseline blood eosinophil count ≥300 cells·µL^−1^ and in the overall patient population

	**Patients with baseline blood eosinophil count** **≥300** **cells·µL^−1^**		**Total patients** ^#^	
	**Benralizumab**	**Placebo**	**Benralizumab**	**Placebo**
**Subjects, n**	448	465	806	806
**Age, years**	49.2±12.7	49.4±12.9	49.8±12.8	50.5±13.0
**Female sex**	277 (61.8)	295 (63.4)	514 (63.8)	517 (64.1)
**Age at diagnosis, years**	30.9±17.9	30.0±18.5	30.7±18.5	30.7±18.9
**Number of previous exacerbations·year^−1^**				
2	269 (60.0)	270 (58.1)	516 (64.0)	503 (62.4)
3	102 (22.8)	106 (22.8)	165 (20.5)	161 (20.0)
4	36 (8.0)	40 (8.6)	57 (7.1)	64 (7.9)
≥5	41 (9.2)	49 (10.5)	67 (8.3)	78 (9.7)
**Use of maintenance OCS**	67 (15.0)	67 (14.4)	114 (14.1)	109 (13.5)
**FEV_1_, % predicted**	56.9±14.8	56.0±14.6	56.4±14.8	56.5±15.3
**FVC, % predicted**	77.2±15.4	76.1±15.3	76.4±15.6	75.9±16.0
**FEV_1_/FVC, %**	59.5±12.6	59.4±12.4	59.7±12.8	60.1±12.9
**PEF, L·min^−1^**	281.6±103.4	268.0±106.3	271.8±100.5	266.5±103.2
**ACQ-6 score**	2.80±0.90	2.86±0.97	2.79±0.90	2.79±0.94
**AQLQ score**	3.92±1.01	3.91±1.04	3.95±1.03	3.96±1.03
**Blood eosinophil count, cells·µL^−1^**	520 (300–2870)	530 (300–4150)	330 (5–2870)	360 (5–4150)
**Daily ICS/LABA dosage**				
High-dose ICS^¶^			737 (91.4)	746 (92.6)
Medium-dose ICS^+^			69 (8.6)	60 (7.4)

Data are presented as mean±sd, median (range) or n (%), unless otherwise indicated. OCS: oral corticosteroids; FEV_1_: forced expiratory volume in one second; FVC: forced vital capacity; PEF: peak expiratory flow; ACQ-6: 6-item Asthma Control Questionnaire; AQLQ: Asthma Quality of Life Questionnaire; ICS: inhaled corticosteroid; LABA: long-acting β_2_-agonist. ^#^: includes patients with baseline blood eosinophil count <300 cells·µL^−1^; ^¶^: >250 µg·day^−1^; ^+^: ≥500 µg·day^−1^.

Of the patients with baseline blood eosinophil counts ≥300 cells·µL^−1^, 63% were female and the mean age was approximately 49 years, with the first asthma diagnosis at approximately 30 years. 59% of patients had two exacerbations, 23% had three, 8% had four and 10% had five or more exacerbations in the previous year; around 15% of patients received maintenance OCS therapy. The median baseline blood eosinophil count was 520 cells·µL^−1^ for those receiving benralizumab and 530 cells·µL^−1^ for those receiving placebo. 92% of patients were receiving high-dose ICS-containing therapies (table 1).

### Event rate, event duration and time to first event in patients with baseline blood eosinophil counts ≥300 cells·µL^−1^

Among patients with baseline blood eosinophil counts ≥300 cells·µL^−1^, benralizumab reduced CompEx AERs *versus* placebo by 39% (1.57 *versus* 2.57; risk ratio 0.61, 95% CI 0.53–0.70, p<0.001). The treatment effect and event rates were similar for SevEx and AWEs, but the higher event rates and similar treatment effect gave tighter CIs for CompEx compared with SevEx alone (supplementary figure S1). The higher power of CompEx allowed trends in treatment effects to be detected earlier compared with SevEx and AWEs. Benralizumab reduced the risk of SevEx by 40% (0.94 *versus* 1.55; risk ratio 0.60, 95% CI 0.52–0.70; p<0.001) and the risk of AWEs by 41% (0.92 *versus* 1.57; risk ratio 0.59, 95% CI 0.48–0.72; p<0.001). Similar reductions in risk of SevEx and AWEs were also seen when data were analysed separately for the individual studies (SIROCCO and CALIMA) (supplementary figure S1).

The duration of events reported tended to be shorter in patients receiving benralizumab than placebo and this was seen consistently for all three end-points (table 2). This was statistically significant for AWE and CompEx, but not for SevEx (Wilcoxon test).

**TABLE 2:** Duration of CompEx, SevEx and AWE events with benralizumab and placebo in patients aged ≥16 years with a baseline blood eosinophil count ≥300 cells·µL^−1^ in the SIROCCO and CALIMA studies (pooled data)

	**Benralizumab**			**Placebo**		
	**CompEx**	**SevEx**	**AWE**	**CompEx**	**SevEx**	**AWE**
**Patients,**^#^ **n**	410	313	234	523	425	331
**Events per patient, mean**	2.3	1.8	2.3	3.2	2.4	3.0
**Percentage of days with an event** **^¶^**						
Mean±sd	6.9±11.4	5.3±6.9	5.3±11.4	8.4±10.8	6.5±7.9	5.6±9.7
Median (range)	2.8 (0.5–90.1)	2.9 (0.8–61.5)	1.4 (0.5–90.1)	4.7 (0.5–75.1)	4.1 (0.3–64.4)	1.7 (0.5–74.9)
**Duration of events, days**						
Mean±sd	24.6±42.2	17.0±19.8	20.5±45.9	33.1±45.4	22.2±26.2	25.9±46.6
Median (range)	10.5 (2.0–317.0)	10.0 (1.0–206.0)	5.0 (2.0–315.0)	17.0 (2.0–278.0)	15.0 (1.0–217.0)	6.0 (2.0–272.0)

CompEx: composite end-point for exacerbations; SevEx: severe exacerbations; AWE: acute worsening events. ^#^: summary statistics are presented for those patients who experienced an event (*i.e.* those without an event are excluded); ^¶^: calculated as a percentage of the total days at risk (of an event) in the study, for each patient.

**FIGURE 1:**
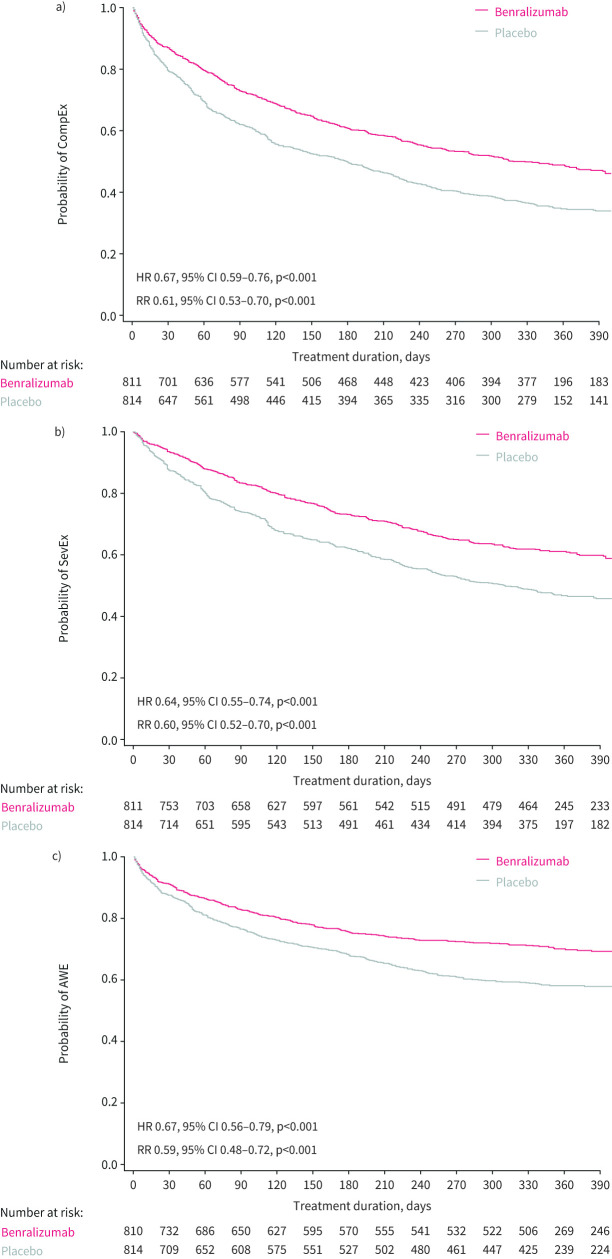
Kaplan–Meier plot for time to first event with benralizumab *versus* placebo for a) composite end-point for exacerbations (CompEx), b) severe exacerbations (SevEx) and c) acute worsening events (AWEs) for all patients aged ≥16 years and a blood eosinophil count ≥300 cells·μL^−1^ from the SIROCCO and CALIMA studies (pooled data). HR: hazard ratio; RR: risk ratio.

Benralizumab was associated with a longer time to first event than placebo for CompEx (HR 0.67, 95% CI 0.59–0.76, p<0.001), SevEx (HR 0.64, 95% CI 0.55–0.74, p<0.001) and AWEs (HR 0.67, 95% CI 0.56–0.79, p<0.001). These trends appeared earlier for CompEx compared with SevEx and AWEs in the Kaplan–Meier curves for the pooled population (figure 1) and per study (supplementary figure S2).

### Predictive value of blood eosinophil counts (all patients)

**FIGURE 2:**
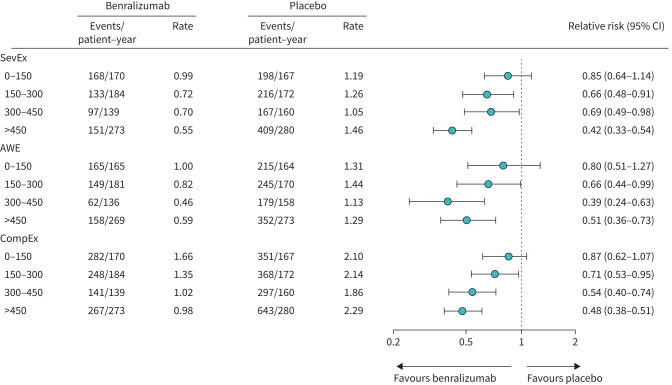
Event rate with benralizumab *versus* placebo for the composite end-point for exacerbations (CompEx), severe exacerbations (SevEx) and acute worsening events (AWEs) for patients aged ≥16 years by baseline blood eosinophil count category (0–150 cells·µL^−1^, 150–300 cells·µL^−1^, 300–450 cells·µL^−1^ and >450 cells·µL^−1^) for SIROCCO and CALIMA studies (pooled data). A restricted model was used with only treatment and study as factors.

**FIGURE 3:**
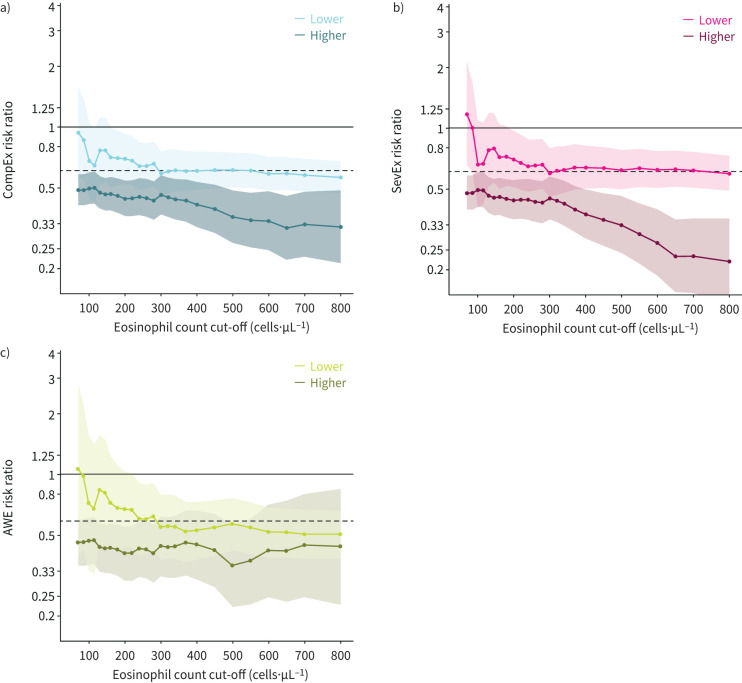
Treatment effect size (risk ratio) by eosinophil count cut-off in the pooled population of patients aged ≥16 years from the SIROCCO and CALIMA studies for a) a composite end-point for exacerbations (CompEx), b) severe exacerbations (SevEx) and c) acute worsening events (AWEs). For each individual eosinophil count plotted, the estimated mean risk ratio and 95% CI are shown for patients with an eosinophil count at or below that value (“lower stratum”; indicated by lines and shading in a lighter tone) and for all patients above the cut-off (“higher stratum”; indicated by lines and shading in a darker tone). For example, in panel a, given an eosinophil count cut-off of 500 cells·µL^−1^, the risk ratio for patients in the higher stratum (those with an eosinophil count >500 cells·µL^−1^) is 0.41 (indicated on the dark blue line). Likewise, the corresponding point for patients with an eosinophil count ≤500 cells·µL^−1^ (lower stratum), shows a risk ratio of 0.68 (on the light blue line). The horizontal dashed line represents the risk ratio for the overall population.

We also analysed event rates and risk ratios from the overall pooled population of patients aged ≥16 years from the SIROCCO and CALIMA studies, across the range of baseline blood eosinophil counts. The treatment effects of benralizumab (as indicated by the risk ratio relative to placebo) on CompEx, SevEx and AWE end-points were consistently higher for patients with higher baseline eosinophil counts (figure 2). For the subgroup of patients with the lowest baseline blood eosinophil counts (<150 cells·µL^−1^), the data were inconclusive because the 95% CI of the risk ratio crosses 1 (figure 2). When analysing the treatment effect size by eosinophil cut-off strata, greater treatment effects were observed for patients in the higher strata (*i.e.* above a given cut-off) than those in the lower strata, irrespective of the eosinophil count cut-off used (figure 3); however, these analyses included low numbers of patients. Furthermore, for CompEx and SevEx, the higher the eosinophil cut-off, the larger the treatment effect within a stratum (figure 3a, b); this effect was less evident within the higher stratum for AWEs (figure 3c).

## Discussion

This *post hoc* analysis of pooled data from SIROCCO and CALIMA provides further evidence for the use of CompEx as an end-point that extends beyond SevEx events, as traditionally measured in clinical trials, by also capturing clinically meaningful worsening in asthma (AWEs). In this subgroup of patients aged ≥16 years with baseline blood eosinophil counts ≥300 cells·µL^−1^ and a history of exacerbations in the prior year, benralizumab improved event rates and time-to-first event *versus* placebo for CompEx and its individual components to a similar extent. Our results suggest that CompEx shows a higher power with tighter CIs compared with SevEx or AWE alone, owing to the higher frequency of events. This higher frequency of events also means that CompEx will have greater statistical power if the treatment difference seen with SevEx or AWE is preserved, allowing trends to be detected slightly earlier or in shorter studies. Similar findings were observed in the individual studies (SIROCCO and CALIMA).

It is well known that both blood and sputum eosinophil counts are important predictors of asthma exacerbations [9, 10, 18]. An association between baseline blood eosinophil counts and exacerbation frequency has previously been observed in a clinical database study, which reported that baseline eosinophil counts >400 cells·µL^−1^ increased the likelihood of two or more severe exacerbations by >1.4-fold [9]. In the present analysis, patients with blood eosinophil counts above any selected cut-off point experienced a substantially greater treatment effect than patients below this same cut-off point. An enhanced treatment effect of benralizumab in patients with high eosinophil counts has been previously described for SevEx [14] and in our *post hoc* analysis. Similar findings were also observed with CompEx: the higher the eosinophil count, the greater the treatment effect of benralizumab. In patients with lower eosinophil counts, there was greater variability in AWEs, and the association with treatment effect was less pronounced. This may be explained by there being fewer events in this population, and the threshold for reporting SevEx or any other trigger of rescue short-acting β-agonist use is also likely to be highly variable. Moreover, a single blood eosinophil count value is not predictive of the eosinophilic type, as there may be some variability in phenotypic expression in patients over time.

Previous analyses of pooled data from the SIROCCO and CALIMA trials showed that characteristics such as adult asthma onset, OCS use, nasal polyposis and eosinophil count ≥300 cells·µL^−1^ are predictive of response to benralizumab treatment [19, 20]. While our analysis suggests that benralizumab is most effective in patients with severe, uncontrolled asthma with higher blood eosinophil counts, we also show that efficacy can be observed in those patients with eosinophil counts <300 cells·µL^−1^ and ≥150 cells·µL^−1^. This is consistent with previous analyses of exacerbation rates and a number of other secondary end-points in the SIROCCO and CALIMA clinical trials for patients with baseline eosinophil counts <300 cells·µL^−1^ [19] and for those with eosinophil counts ≥150 cells·µL^−1^ [21]. In the randomised, controlled ZONDA [22] and PONENTE [23] clinical trials and the ANDHI in Practice sub-study [24], benralizumab significantly reduced oral glucocorticoid doses from baseline levels in patients with baseline blood eosinophil counts ≥150 cells·µL^−1^. These findings are also supported by real-world evidence showing reduced exacerbation rate and oral glucocorticoid dose in patients with severe, uncontrolled eosinophilic asthma, including those with baseline eosinophil counts <300 cells·µL^−1^ [25]. This supports the importance of identifying the eosinophilic phenotype of patients, through blood eosinophilia and/or patient characteristics that may predict the response to eosinophil-targeted therapy.

A major strength of this analysis was the use of a comprehensive dataset with a large number of patients from two trials with similar study designs and outcomes, to further evaluate the CompEx end-point. However, the findings are limited by the *post hoc* nature of the analyses. For example, the study was descriptive and statistical testing for differences in the duration of events between treatment groups was conducted *post hoc*. While our findings suggest shorter duration of events in the benralizumab group than the placebo group using all three end-points, this would need to be confirmed by further studies. Additionally, in the two studies included in the analysis, the frequency of SevEx was high while the frequency of AWEs was only modest, resulting in a larger overlap between SevEx and CompEx compared with previous studies. Consequently, the benefit of using CompEx over SevEx was less evident here. CompEx requires the capture of more patient-reported outcomes than SevEx alone, which increases patient burden, because of reliance on appropriate use of diaries and patient compliance when filling out diary cards. This additional patient burden may be overcome in the future because of advances in digital healthcare solutions. Furthermore, the increased burden from use of CompEx must be balanced against the possibility of smaller trial populations or shorter trial durations owing to its increased power to detect differences between treatment groups. Further in-depth analysis of CompEx variables to better understand the additional value that CompEx brings beyond trial length and size is ongoing in the phase 4 BenRex study (ClinicalTrials.gov: NCT04102800).

### Conclusions

Benralizumab reduced the risk of CompEx events in patients with severe, uncontrolled asthma and a history of exacerbations, with a similar treatment effect on SevEx and AWEs across a range of blood eosinophil counts. Baseline blood eosinophil count was predictive of the treatment effect of benralizumab on CompEx and its individual components, with higher eosinophil counts predictive of greater treatment effect. Our findings indicate that the use of the CompEx end-point (combining AWEs and SevEx) may have value beyond increasing the number of events observed by also supporting the evaluation of benralizumab and other novel drugs based on objectively defined clinical variables such as PEF. Improved longitudinal quantification of changes to asthma-relevant variables could, in turn, contribute to improved clinical management of patients and thus to potentially reduced healthcare utilisation costs.

## Supplementary material

10.1183/23120541.01025-2023.Supp1 **Please note:** supplementary material is not edited by the Editorial Office, and is uploaded as it has been supplied by the author.
Supplementary material 01025-2023.SUPPLEMENT
