# Supplementary figures and images for: Use of CompEx in eosinophilic patients with severe, uncontrolled asthma on benralizumab
Source: ERJ Open Res. 2024 Mar 18;10(2):01025-2023. doi: 10.1183/23120541.01025-2023 (PMC10945385; doi:10.1183/23120541.01025-2023)

a. CompEx

SIROCCO

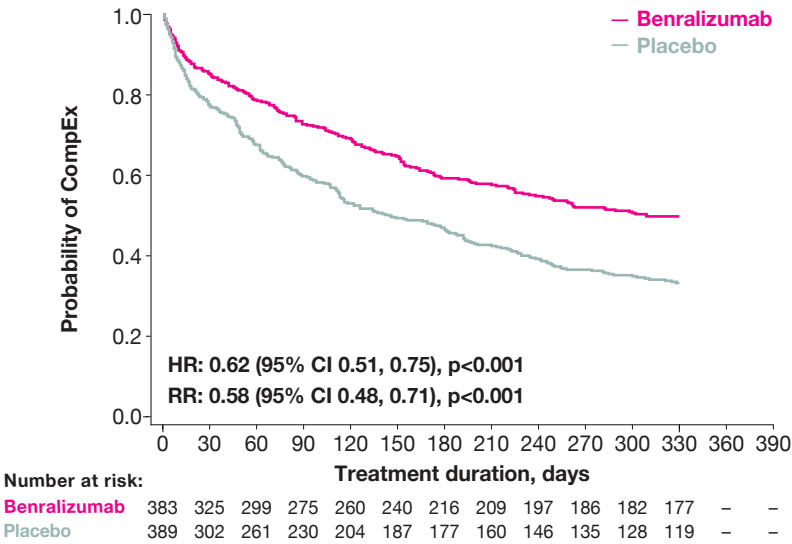

CALIMA

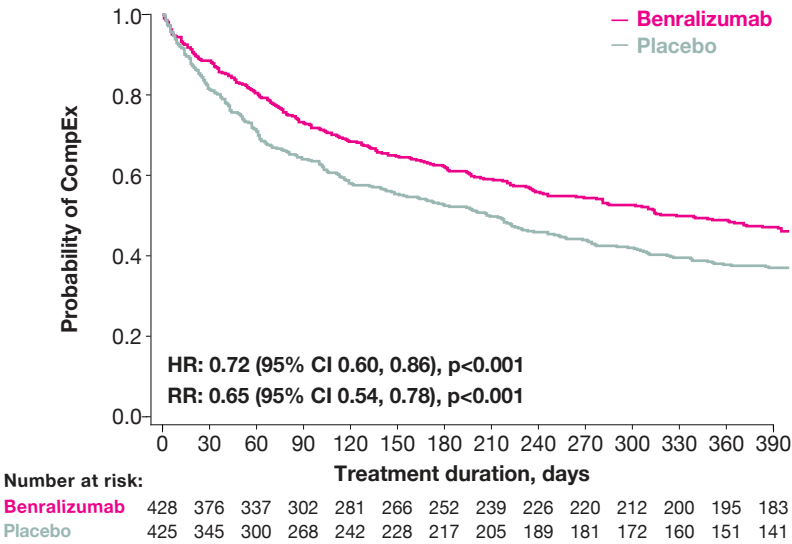

b. SevEx

SIROCCO

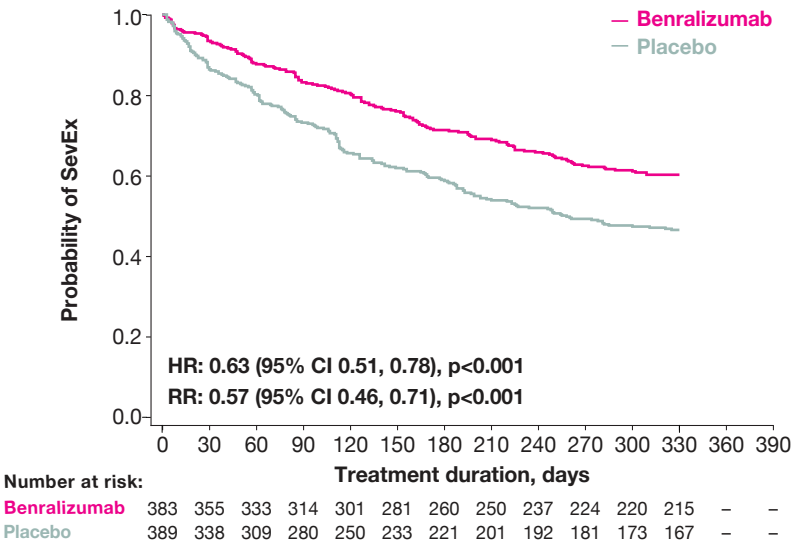

CALIMA

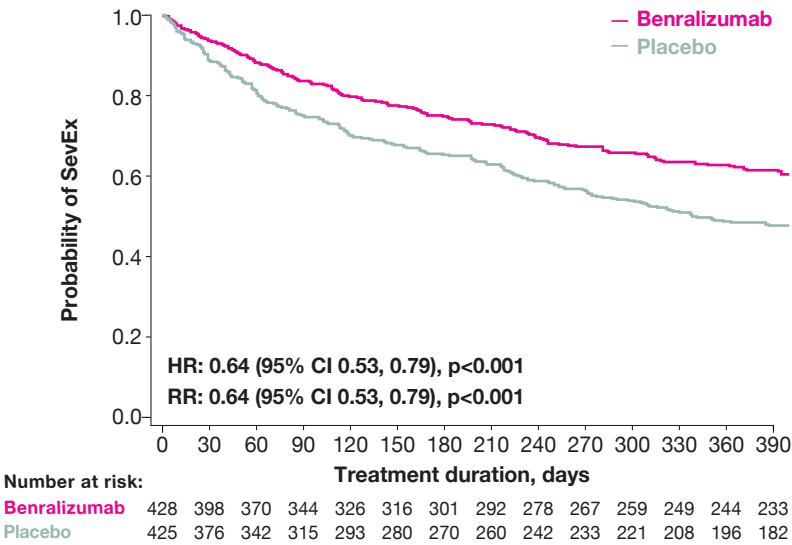

c. AWE

SIROCCO

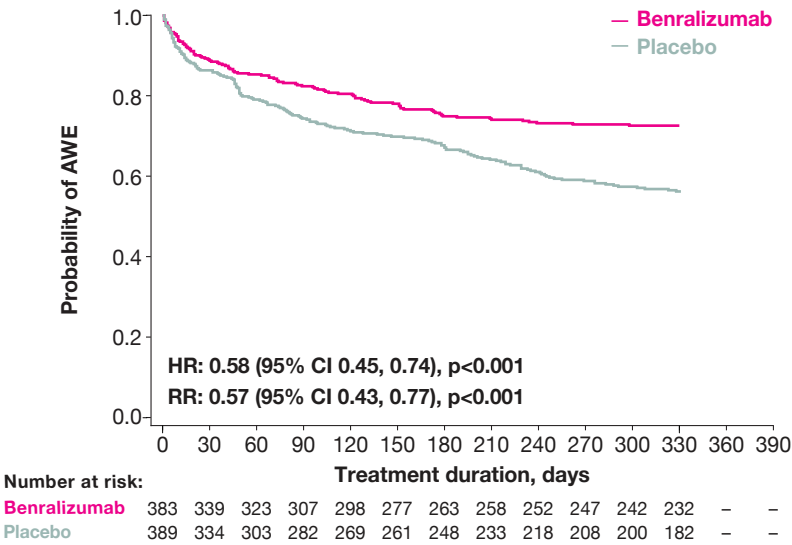

CALIMA

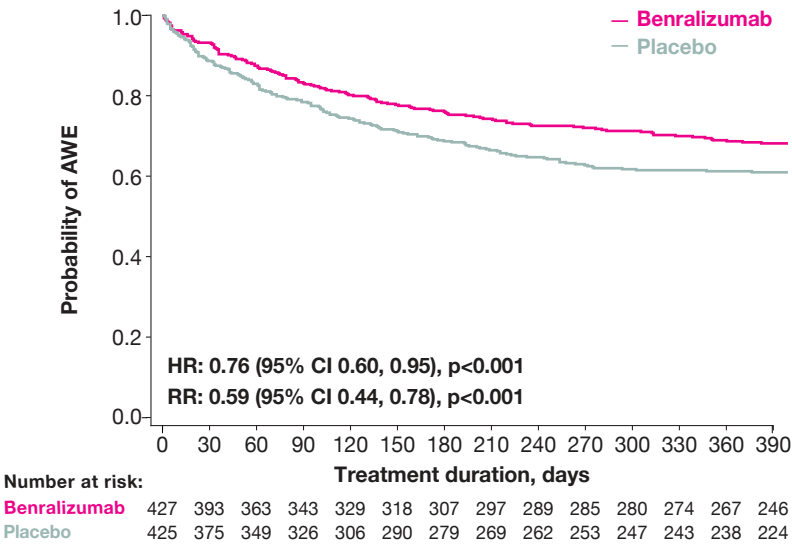

Supplement: Supplementary file 1 [file 01025-2023.SUPPLEMENT.pdf]
